# A *Sporolactobacillus*-, *Clostridium*-, and *Paenibacillus*- Dominant Microbial Consortium Improved Anaerobic RDX Detoxification by Starch Addition

**DOI:** 10.4014/jmb.1910.10034

**Published:** 2020-03-09

**Authors:** Muhammad Imran Khan, Keunje Yoo, Seonghoon Kim, Sardar Alam Cheema, Safdar Bashir, Joonhong Park

**Affiliations:** 1 Department of Civil and Environmental Engineering, College of Engineering, Yonsei University, Seoul 03722, Republic of Korea; 2 Institute of Soil and Environmental Sciences, University of Agriculture, Faisalabad 38040, Pakistan; 3 Department of Agronomy, University of Agriculture, Faisalabad 8040, Pakistan; 4 Department of Isotope Biogeochemistry, Helmholtz Centre for Environmental Research- UFZ, 0318 Leipzig, Germany; 5 Department of Environmental Engineering, College of Engineering, Korea Maritime and Ocean University, Busan 49112, Republic of Korea

**Keywords:** Bioremediation, explosives, MiSeq, starch, *Sporlactobacillus*

## Abstract

In the present study, an anaerobic microbial consortium for the degradation of hexahydro-1,3,5-trinitro-1,3,5-triazine (RDX) was selectively enriched with the co-addition of RDX and starch under nitrogen-deficient conditions. Microbial growth and anaerobic RDX biodegradation were effectively enhanced by the co-addition of RDX and starch, which resulted in increased RDX biotransformation to nitroso-derivatives at a greater specific degradation rate than those for previously reported anaerobic RDX-degrading bacteria (isolates). The accumulation of the most toxic RDX degradation intermediate (MNX [hexahydro-1-nitroso-3,5-dinitro-1,3,5-triazine]) was significantly reduced by starch addition, suggesting improved RDX detoxification by the co-addition of RDX and starch. The subsequent MiSeq sequencing that targeted the bacterial 16S rRNA gene revealed that the *Sporolactobacillus*, *Clostridium*, and *Paenibacillus* populations were involved in the enhanced anaerobic RDX degradation. These results suggest that these three bacterial populations are important for anaerobic RDX degradation and detoxification. The findings from this work imply that the *Sporolactobacillus*, *Clostridium*, and *Paenibacillus* dominant microbial consortium may be valuable for the development of bioremediation resources for RDX-contaminated environments.

## Introduction

Hexahydro-1,3,5-trinitro-1,3,5-triazine (RDX) is one of the most commonly used explosives in the world [1]. The global manufacturing, storage, detonation, and disposal of this explosive compound have caused widespread contamination of soil [2, 3]. RDX migrates through soil and potentially causes groundwater contamination [4-6] owing to its weak sorption affinity for soil and fairly high solubility in water (60 mg l^-1^ at 20°C) [7]. RDX is classified as a class C carcinogen, and it exhibits toxic properties to organisms even at low concentrations [8, 9]. Because of the high degree of pollution exposure and toxicity of RDX, the removal of RDX from polluted environments is of vital importance for the protection of human health and ecological systems against explosive contamination.

Microorganisms have been shown to be able to biodegrade RDX under aerobic and anaerobic conditions via different degradation pathways [10-11]. Under aerobic conditions, one nitro group of the RDX is removed (via denitration-hydration step) before the destabilization and cleavage of ring, resulting in the formation of 4-nitro-2,4-diazabutanal (NDAB) or methylenedinitramine (MEDINA), formaldehyde, and carbon dioxide [10, 11]. Under anaerobic conditions, RDX is degraded via sequential nitro-reduction to produce hexahydro-1-nitroso-3,5-dinitro-1,3,5-triazine (MNX), hexahydro-1,3-dinitroso-5-nitro-1,3,5-triazine (DNX) and hexahydro-1,3,5-trinitroso-1,3,5-triazine (TNX) before ring cleavage. The MNX may further denitrosate or denitrate and after ring cleavage produce MEDINA or NDAB [or 4-nitoso-2,4-diazabutanal (NO-NDAB)]. The direct ring cleavage of RDX produces MEDINA and bis(hydroxymethyl)nitramine (BHNA), followed by formation of nitrous oxide and formaldehyde as end products [10, 11].

Although aerobic microbial degradation of RDX has recently been regarded as a promising biological process to clean up explosive-contaminated sites [10-18], the use of aerobic RDX degradation in contaminated sites might be limited because of a low saturation level of dissolved oxygen (DO) and the cost-ineffective means of supplying DO [8, 11], particularly in subsurface environments. When DO supply is limited, anaerobic microbial degradation of RDX is regarded as a feasible biological alternative for bioremediation of explosive-contaminated sites [8, 11, 20, 21].

For anaerobic degradation of RDX, an external carbon source is often added to provide enhanced reducing conditions [4] or to stimulate the RDX degradation and growth of anaerobic RDX-degrading microbes [22-26]. In the case of bacterial isolates, simple carbon sources such as glucose [24, 27], acetate [28, 29], succinate [30] and ethanol [23, 28] have been typically used for stimulation of microbial degradation of RDX. However, for mixed culture conditions or consortia, the use of simple sources of carbon may not work well because simple carbon sources may be preferentially used by fast-growing non-degradative bacteria instead of relatively slow-growing RDX-degrading bacteria [31, 32]. To circumvent this problem, slow-releasing sources of carbon such as starch and molasses have often been used to maintain a high degree of degradative activity and population diversity in microbial mixed-culture or community conditions [4, 33, 34].

Starch is an effective stimulant for enriching RDX-degrading consortia because of its widespread availability, cost-effectiveness, easy biodegradability, and complex nature [28]. Our previous study revealed that co-addition of RDX and starch could enhance RDX degradation and detoxification under aerobic conditions [34], and the 16S rRNA gene amplicon pyrosequencing identified the involvement of a *Rhizobium* population in the enhanced aerobic RDX degradation and detoxification by starch addition. However, the effects of starch addition on anaerobic RDX degradation/detoxification and population dynamics have yet to be examined.

In this work, starch was used in the selective cultivation of a novel anaerobic microbial consortium capable of RDX detoxification. The specific research objectives were (i) to study the impacts of starch addition on the formation of intermediates and specific biodegradation rates of anaerobic RDX degradation under no fixed-nitrogen conditions and (ii) to explore its effects on the bacterial communities and identify bacterial populations related to anaerobic RDX detoxification.

## Materials and Methods

### Chemicals

RDX (purity > 99%) was obtained from AccuStandard, Inc. (USA). HPLC-grade methanol, isopropyl alcohol, acetone, and acetonitrile were obtained from J. T. Baker (USA). Starch (soluble) was obtained from Junsei Chemicals Co. Ltd. (Japan). Water, used for the HPLC mobile phase and the aqueous growth medium, was purified using a Compact Ultrapure Water System and an EASYpure Reverse Osmosis System obtained from Barnstead/Thermolyne (USA).

### Microcosm Experiments

Anaerobic incubation experiments were carried out using 160-ml glass serum bottles containing 150 ml of aqueous growth medium. The growth medium was comprised of as follows (in grams per liter): KCl (0.1), NaH_2_PO_4_·H_2_O (0.6), NaHCO_3_ (2.5), 0.5 ml of 1 mM Na_2_SeO_4_, and modified Wolfe’s mineral and vitamin mixtures (each 10 ml per liter) [35]. Starch (at different concentrations, *i.e.* 0, 2.5, and 5.0 g l^-1^) was added as a slow-releasing external source of carbon in this study. After 1 h of flushing with oxygen-free nitrogen gas, microcosm bottles were sealed and autoclaved (45 min at 121°C). RDX was added to the microcosm bottles using acetone as a carrier solvent and bottles were shaken to dissolve RDX in liquid growth medium. To evaporate the acetone, bottles were placed in an anoxic chamber (Coy Laboratory Products Inc., USA) for 8 h. Approximately 10 ml of liquid medium was removed from the prepared bottle and was added into a clean sterile falcon tube carrying 5 g of soil collected from a military shooting site at Darokdae (Korea).

Falcon tubes were shaken and swirled for the extraction of inoculum. Extractants (carrying inocula) were added back to the representing bottle, after settling larger soil particles. Sterile controls were also prepared to assess abiotic abasement of RDX. In the control microcosm, bottles were autoclaved again after addition of inocula, and finally RDX was added in the autoclaved bottles. Microcosm bottles were completely wrapped with aluminum foil to avoid photo-degradation of RDX [36]. The final pH was 7.1 ± 0.2. The details of different treatments used in first and each subsequent sub-culture experiment are provided in Table S1, and all experiments were done in triplicate. The microcosm bottles were incubated at 25.0 ± 0.5°C in the dark without shaking. Periodic samples were taken for analysis using disposable syringes and 21-gauge needles.

After the 1^st^ enrichment experiment, a subculture experiment (*i.e.*, 2^nd^ enrichment) was conducted to affirm the degradative potential of growing culture and screen RDX-degrading consortia. Approximately 15.0 ml of grown culture from the 1^st^ enrichment was transferred to 135 ml of freshly prepared sterile anoxic liquid growth medium containing RDX using disposable syringes and 21-gauge needles, and the resultant microcosm was named the 2^nd^ enrichment. Similarly, 150 ml of 3^rd^ enrichment microcosm was prepared by receiving 15.0 ml of culture from the 2^nd^ enrichment. All microcosms were incubated without shaking at 25.0 ± 0.5°C in the dark, and they were sampled periodically to monitor RDX degradation and microbial growth. Among each three replicates, the one with the best biodegradation was chosen for further transfer and estimation of biodegradation rates.

### Specific Rates of RDX Degradation

Specific biodegradation rates (R) of RDX were calculated using the following equation:

(1) $$R=\frac{\left(C_{n}-C_{\mathrm{n-1}}\right)}{\left(t_{n}-t_{\mathrm{n-1}}\right)}\times \frac{1}{{\mathrm{OD}}_{\mathrm{av}}}$$

where *t_n_* and *t_n-1_* represent two continuous sampling times, C_n_ and C_n-1_ represent the concentrations of RDX in the microcosms at the matching sampling times, and *OD_av_* represents the average OD values between *t_n_* and *t_n-1_*.

### Analytical Methods

RDX and its biotransformation intermediates, such as MNX, DNX, and TNX, were analyzed using HPLC (Agilent Technologies 1200 series, USA) at 230 nm following the EPA Method 8330 [37, 38] with slight modifications as described in Khan *et al*. [34]. For quantification of microbial growth, optical density (OD) at 600 nm was measured using a spectrophotometer (UV/VIS) (Mecasys Co. Ltd., Korea).

### Statistical Anaylses

Data were analyzed using the SPSS statistical package, version 16.0 (SPSS Inc., Chicago, IL) for Windows [39]. ANOVA was applied to find differences between different treatments and controls (*p* < 0.05).

### DNA Extraction, PCR Amplification, MiSeq Sequencing

Extraction of total genomic DNA from the soil sample (initially used for the 1^st^ enrichment) and from selected microcosms (amended with different concentrations of starch) of 3^rd^ enrichment (after 26 days of incubation) was done using a Soil DNA Extraction Kit (MOBIO, CA) following the manufacturer’s guidelines. Bacterial 16S rRNA genes were PCR amplified and V3 and V4 regions were targeted using the forward primer (5′-TCGTCGGCA-GCGTCAGATGT-GTATAAGAGACAGCCTACGGGNGGCWGCAG-3′) and reverse primer (5′-GTCTC-GTGGGCTCGGAGATGTGTATAAGAGACAGGACTACHVGGGTA-TCTAATCC-3′) [40]. Each PCR reaction (for a total volume of 25 μl) contained 2.5 μl of microbial DNA (5 ng/ μl), 12.5 μl of 2x KAPA HiFi HotStart ReadyMix, and 5 μl of each primer (1 μM). Amplification occurred in a GeneAmp PCR System 9600 Thermal Cycler (Applied Biosystems, USA) and amplification conditions are: an initial denaturation step at 94°C for 3 min, followed by 25 cycles of denaturation at 95°C for 30 sec, annealing at 55°C for 30 sec, and extension phase at 72°C for 30 sec, and subsequently followed by a final extension at 72°C for 5 min. The PCR purification was performed using the MinElute PCR Purification Kit (Qiagen, USA) [41]. Amplicon sequencing was conducted by Macrogen (Korea) on the Illumina MiSeq platform. Analysis of sequences was carried out according to the protocol suggested by Kozich *et al*. [42] using Mothur (v. 1.31). A phylogenetic tree analysis was conducted for comparison of the sequences (obtained in this study) with the 16S rRNA gene sequences existing in the GenBank through BLAST: http://www.ncbi.nlm.nih.gov/BLAST/. The MUSCLE was used for sequence alignment [43], and MEGA X software program was used to build a phylogenetic tree following the method described by Khan *et al*. [34]. The representative nucleotide sequences obtained in this study were deposited in the GenBank under the accession numbers KR779830- KR779845.

## Results

### Impact of Starch Addition on Anaerobic RDX Degradation

**Fig. 1:**
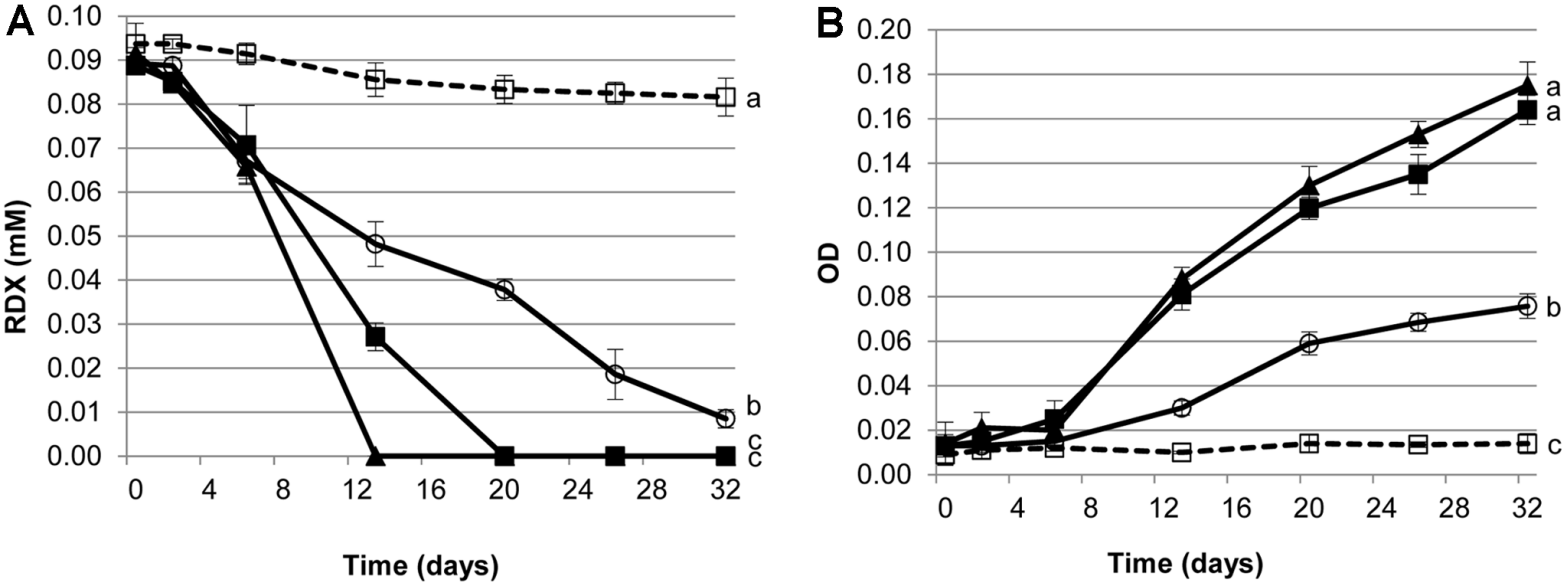
Anaerobic biodegradation of RDX (A) and microbial growth (B) in the 3^rd^ (last) enrichment experiment. The symbols indicate autoclaved control microcosm with RDX in the absence of starch (--□--), microcosm with RDX in the absence of starch (-○-), microcosm with RDX in the presence of 2.5 g/L of starch (-▲-), and microcosm with RDX in the presence of 5.0 g/l of starch (-■-). Each error bar represents one standard deviation of three replicates. Variants with the same lower-case letter are not significantly different at *p* < 0.05. (OD, optical density at 600 nm).

**Fig. 2:**
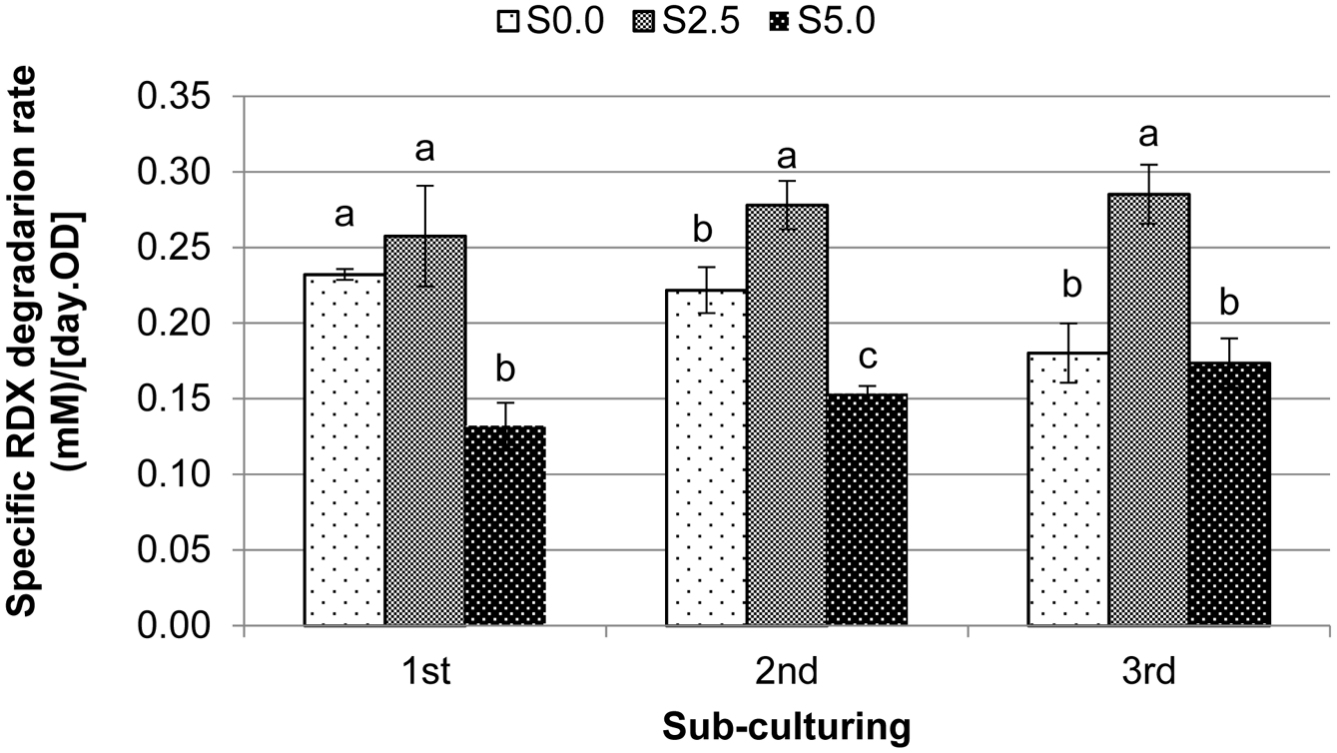
Specific rates of anaerobic RDX degradation in response to sub-culturing and starch addition. *S0.0* indicates the microcosm with RDX in the absence of starch, *S2.5* is the microcosm with RDX in the presence of 2.5 g/l of starch, and S5.0 is the microcosm with RDX in the presence of 5.0 g/l of starch. Variants with the same lower-case letter are not significantly different at *p* < 0.05. Each error bar represents one standard deviation of three replicates.

Significant disappearance of RDX was observed in the live microcosms (*solid line*) compared with the abiotic losses detected in the autoclaved control experiments (*dashed line*) (Fig. 1A). Interestingly, a significant amount of RDX was degraded in the live microcosms in the absence of starch (*blank circle*). The co-addition of starch with RDX enhanced the growth-associated biodegradation of RDX vs. that for the no-starch RDX-induced consortium (S0.0) (Fig. 1). When 2.5 g/l of starch was amended, the greatest specific rate of RDX biodegradation was observed among the tested microcosms; a higher starch concentration (5.0 g/l) resulted in a reduced specific degradation rate (Fig. 2). In the following 2^nd^ and 3^rd^ sub-culture experiments, specific RDX biodegradation rates for S2.5 and S5.0 were further increased (*p* < 0.147 for S2.5 and *p* < 0.061 for S5.0). In the no-starch RDX-induced microcosms (S0.0), however, the specific rate of RDX biodegradation further decreased in the subsequent sub-culturing experiments (*p* < 0.037), indicating an increased loss of RDX degradation activity in the no-starch and RDX-induced consortium during the sub-culturing. This might be due to the loss of active biomass resulting from sub-culturing. The anaerobic RDX-degrading microbial consortium obtained from the 3^rd^ (the final) sub-culture experiments was named IK. Specific rates of anaerobic RDX degradation by the IK consortium in the presence (S2.5) and absence of starch (S0.0) were greater (*p* < 0.01) than those for most of the known anaerobic RDX-degrading bacteria (Table 1).

### Impact of Starch Addition on Degradation Intermediates

**Fig. 3:**
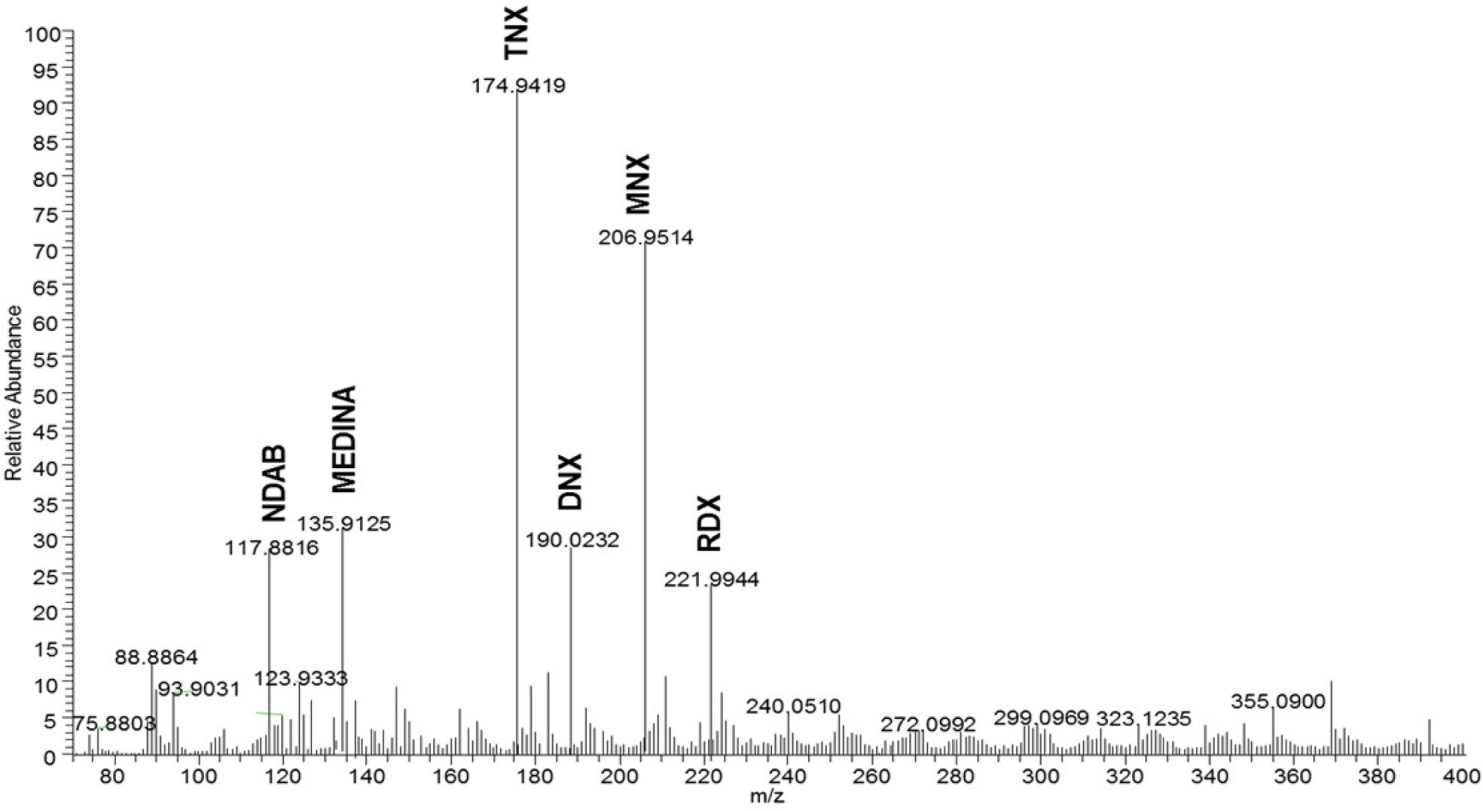
Mass spectra (LC/MS) of the metabolites formed during microbial degradation of RDX. The MNX, DNX, TNX, MEDINA, and NDAB indicate hexahydro-1-nitroso-3,5-dinitro-1,3,5-triazine, hexahydro-1,3-dinitroso-5-nitro- 1,3,5-triazine, hexahydro-1,3,5-trinitroso-1,3,5-triazine, methylenedenitramine, and 4-nitro-2,4-diazabutanal respectively.

**Fig. 4:**
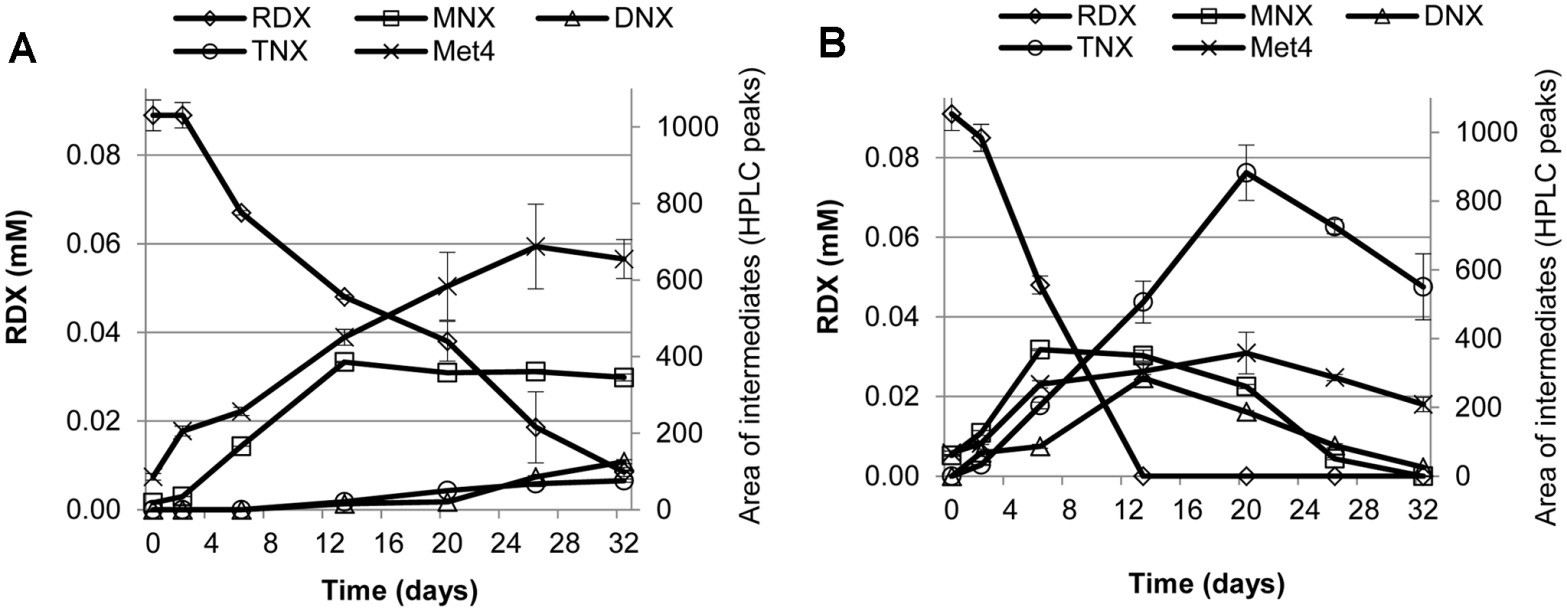
Time-course changes in anaerobic RDX degradation intermediates in *S0.0*, the microcosm with RDX in the absence of starch (A) and *S2.5*, the microcosm with RDX in the presence of 2.5 g/l of starch (B) in the 3^rd^ enrichment experiments. Each error bar represents one standard deviation of three replicates. [Met4, metabolite 4 (suspected to be MEDINA or NDAB)].

Through sequential nitro-reduction, anaerobic microbial degradation of RDX produces MNX, DNX and TNX before ring cleavage; however, the direct ring cleavage of RDX yields MEDINA and BHNA as degradation intermediates [10]. Moreover, the ring cleavage of MNX may further denitrosate or denitrate to produce MEDINA or NDAB (or even NO-NDAB), respectively [11]. In the present study, the HPLC analysis showed the formation of MNX, DNX, and TNX as the main RDX degradation intermediates in anaerobic RDX-degrading microcosms (Fig. S1). Furthermore, chromatograms of HPLC demonstrated the production of an unknown metabolite (Fig. S1), which was identified as more likely to be MEDINA and/or less likely to be NDAB through LC–MS analysis (Fig. 3). In the no-starch microcosm (S0.0), MNX and MEDINA/NDAB were accumulated throughout the incubation period, and only a small amount of MNX was further transformed to DNX or TNX (Fig. 4A). Interestingly, in the starch-amended microcosms (*i.e.*, S2.5), MNX was completely transformed into DNX and then into TNX. The accumulated TNX was eventually reduced as the incubation time increased (Fig. 4B). All RDX degradation intermediates including TNX and MEDINA/NDAB were completely removed after 75 days of incubation in the starch-amended microcosms (data not shown). However, in the same incubation period, traces of MNX were still detected in the no-starch amended microcosms (data not shown). The results indicate that the degree of RDX detoxification was significantly improved by the addition of starch.

### Anaysis of Microbial Community

MiSeq sequencing targeting the 16S rRNA gene revealed that *Sporolactobacillus* (RCS1–RCS5) and *Clostridium* (RCS6–RCS10) members became predominant in all RDX-degrading microcosms regardless of starch addition. In the S2.5 microcosm that exhibited the greatest RDX degradation rate, *Sporolactobacillus* was the predominant genus group (49.8% of total), followed by *Clostridium* (42.6% of total) (Table 2). Similar results were observed in S5.0 in which the relative abundances of *Sporolactobacillus* and *Clostridium* were 46.2% and 38.4%, respectively. In the S0.0 microcosm, *Clostridium* was dominant (48.2% of the total), followed by *Sporolactobacillus* and *Paenibacillus* (RCS11–RCS12) (31.6% and 11.8% of the total, respectively). The *Sporolactobacillus* and *Clostridium* populations may have been stimulated by RDX alone, and their stimulation was enhanced by the addition of starch. In the case of *Paenibacillus*, its growth-associated RDX degradation may have been stimulated by RDX alone, but their relative abundance was reduced in the presence of starch (Table 2).

**Fig. 5:**
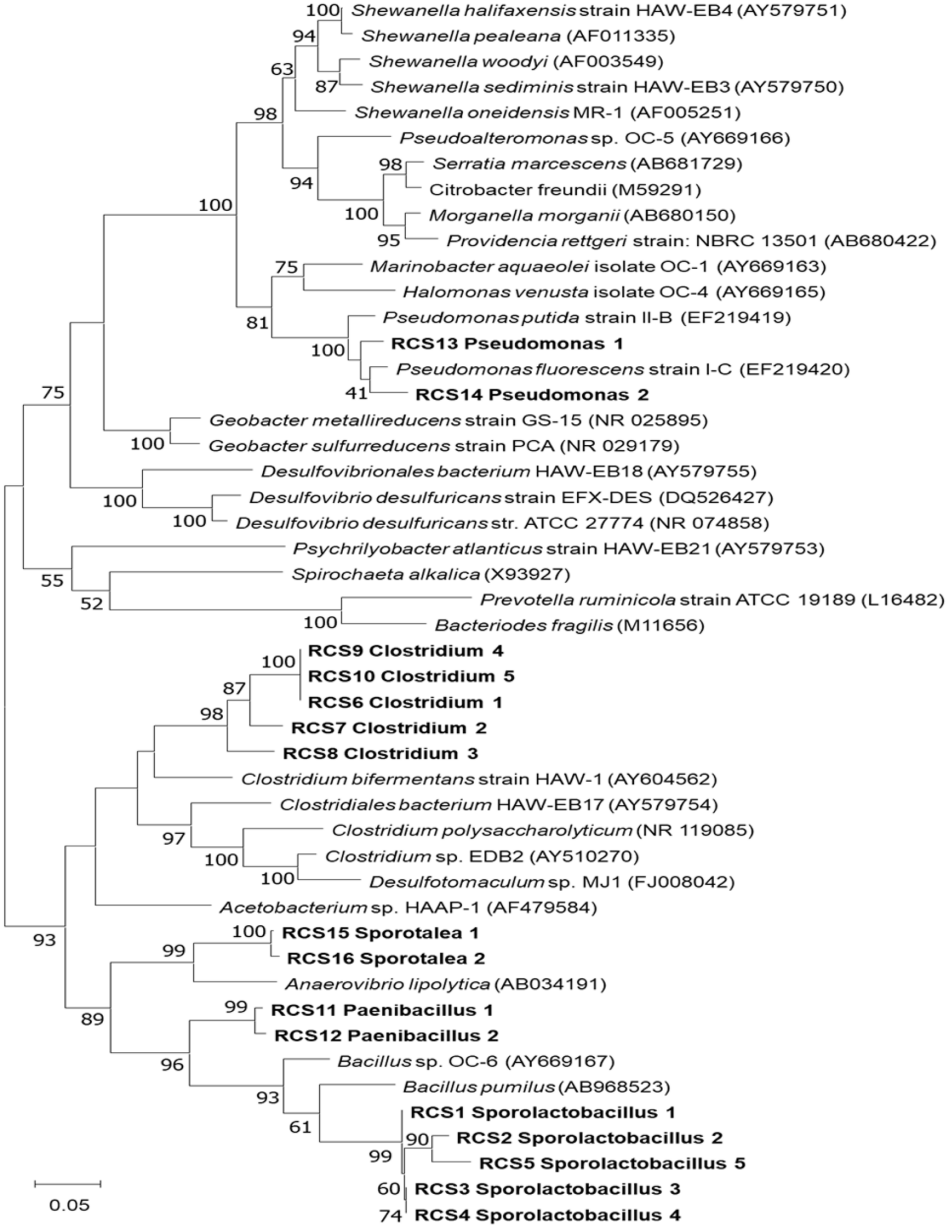
Phylogenetic tree illustrating the distribution of the 16S rRNA sequences (the average size of the aligned and trimmed sequences was 470 bp), which were obtained by MiSeq sequencing (RCS1-RCS16), and their relationship with known anaerobic RDX-degrading bacterial isolates. The numbers beside the nodes indicate the bootstrap values from the bootstrapping test (1,000 replicates). The scale bar represents 16S rRNA sequence divergence.

A phylogenetic analysis was carried out to compare the 16S rRNA gene sequences of the bacterial populations stimulated by RDX with those of previously known RDX-degrading bacteria (Fig. 5). The 16S rRNA sequences of the *Sporolactobacillus* (RCS1–RCS5), *Clostridium* (RCS6–RCS10) and *Paenibacillus* (RCS11–RCS12) members, the relative abundances of which significantly increased during the enrichment with RDX, were found to be phylogenetically distant from the known anaerobic RDX-degrading bacteria (more than 5% dissimilarity). The results suggested that some members of *Sporolactobacillus* (RCS1–RCS5), *Clostridium* (RCS6–RCS10) and *Paenibacillus* (RCS11–RCS12) may be novel anaerobic RDX-degrading bacteria. However, future studies will confirm the novelty of these members.

## Discussion

The present work sought to use the co-addition of RDX and starch to develop anaerobic bacterial consortia for RDX detoxification. Furthermore, the effects of the starch addition on the rates and intermediate products of anaerobic RDX degradation and on the microbial community attributes were explored. An anaerobic RDX-detoxifying bacterial consortium was successfully enriched by the co-addition of RDX and starch, and this mixture exhibited a greater specific degradation rate than those for previously reported anaerobic RDX-degrading bacterial isolates (Table 1) [24,27,44-46]. To the best of our knowledge, this is first study reporting the involvement of the members of three genera including *Sporolactobacillus*, *Clostridium* and *Paenibacillus* together in anaerobic RDX degradation.

In this study, the sequential reduction of the nitro groups of RDX and the formation of its nitroso-derivatives by IK consortium may indicate the involvement of a nitro-reduction pathway in the observed degradation [24, 45]. However, the accumulation of nitroso-derivatives of RDX, particularly MNX is problematic because MNX is known to be the most toxic nitroso-derivatives of RDX [the order of toxicity: MNX > TNX > DNX > RDX] [11, 47]. Interestingly, MNX, the toxic RDX degradation intermediate, was significantly reduced by the addition of starch, showing improved RDX detoxification by the addition of starch. Furthermore, the formation of MEDINA or NDAB may be an indication of the ring cleavage of the nitroso-derivative intermediates [48]. The ring cleavage is considered an important step leading to the more complete degradation or mineralization.

The improvement in RDX detoxification by starch addition may be attributable to the stimulated growth of *Sporolactobacillus* in the microcosm. Because a significant stimulation of its growth (by the co-addition of RDX and starch) (Table 2) was positively correlated with the enhanced degradation of RDX and its nitroso-derivatives (Fig. 4). These possibilities are further supported by the literature reports that *Sporolactobacillus terrae* DSM 11697 [49] and *S. laevolacticus* DSM 442 [50] contain a variety of toxicant degrading enzymes, such as nitroreductases (WP_028982639, WP_023508470), NADPH oxidoreductases (WP_028978040, WP_031263679), dehydrogenases (WP_028983213, WP_023510728), and cytochrome C (WP_037562652, WP_031264216), which are enzymes that potentially degrade RDX and its nitroso-derivatives [11,51-52]. These findings indicate that the RDX detoxification in the anaerobic consortium may have been improved, probably via nitroreductases [51] and dehydrogenases [53] pathways, and these improvements were mainly in the starch–RDX stimulated potential RDX degraders. This present study together with our previous study demonstrated that the addition of starch enhanced the detoxification of RDX into non-toxic or less-toxic degradation products under anaerobic conditions as well as aerobic conditions [34]. Moreover, a significant amount of RDX was degraded in the no-starch live microcosms, suggesting the possible roles of RDX itself as an inducing agent for RDX degradation and a substrate for microbial growth [54]. However, for the validity of this possibility (utilization of RDX as a substrate) further stable isotope probing (SIP) experiments on ^15^N-labeled RDX are required.

The potential RDX degraders that were dominant in the RDX-degrading consortium were found to belong to *Sporolactobacillus*, *Clostridium*, and *Paenibacillus*, which are facultative anaerobic, gram-positive, endospore-forming bacteria [55-57]. *Clostridium* is a well-known anaerobic RDX degrader [8, 27, 58, 59] and is known to carry dehydrogenases [58, 60] and diaphorases [59, 61] that are involved in RDX degradation. There are several reports on the involvement of *Sporolactobacillus* and *Paenibacillus* in anaerobic degradation of octahydro-1,3,5,7-tetranitro-1,3,5,7-tetrazocine (HMX) [47, 62]. However, there has been only few reports regarding the involvement of *Sporolactobacillus* and *Paenibacillus* in anaerobic RDX degradation [63].

In short, an anaerobic bacterial consortium (IK) that can biodegrade RDX was successfully developed through sub-culture techniques with the co-addition of RDX and starch. The developed bacterial consortium was able to biotransform RDX into MNX, DNX and TNX as well as NDAB/MEDINA probably via a ring cleavage with relatively higher degradation rates. Furthermore, the detoxification of RDX by the developed anaerobic consortium was improved by the addition of starch. Taken together with our previous study of starch-enhanced aerobic RDX detoxification, the findings of this study suggest that, regardless of oxygen conditions, the addition of starch could be an effective way to stimulate explosive biodegradation for improving explosive detoxification.

## Supplemental Materials

Supplementary data for this paper are available on-line only at http://jmb.or.kr.

## Figures and Tables

**Table 1:** Comparison of anaerobic RDX biodegradation rates between the known RDX-degrading isolates and IK consortium from this work.

Microbial isolates/consortia	Carbon substrate (g/L)	RDX (mM)	Degradation rate (mM/day)	Specific degradation rate [(mM)/(day.OD)] ^1^	References
*Acetobacterium malicum* strain HAAP-1	*NA*	0.029	0.002	*NA*	[45]
*Anaerovibrio lipolyticus*	Acetate (4.1) + butyrate (1.8) + citrate (1.1)	0.059	0.012	*NA*	[29]
*Citrobacter freundii*	Citrate (0.06)	*NA*	0.007	0.003-0.005	[24]
*Clostridium bifermentans* HAW-1	*NA*	0.104	0.032	0.154-0.172	[44]
*Clostridium polysaccharolyticum*	Acetate (4.1) + butyrate (1.8) + citrate (1.1)	0.074	0.014	*NA*	[29]
*Clostridium* sp. strain EDB2	Glucose (4.5) + lactate (2.25)	0.020	0.010	0.018-0.022	[27]
*Desulfovibrio desulfuricans*	Acetate (4.1) + butyrate (1.8) + citrate (1.1)	0.008	0.002	*NA*	[29]
*Klebsiella pneumoniae strain* SCZ-1	Glucose (1.0)	*NA*	0.009	0.010-0.014	[24]
*Prevotella ruminicola*	Acetate (4.1) + butyrate (1.8) + citrate (1.1)	0.032	0.006	*NA*	[29]
*Pseudomonas fluorescens* I-C	Succinate (0.73)	0.016	0.001	*NA*	[30]
*Pseudomonas putida* II-B	Succinate (0.73)	0.011	0.004	*NA*	[30]
*Shewanella halifaxensis* HAW-EB4	*NA*	0.090	0.004	0.001-0.002	[46]
**IK consortium**	**Starch (2.5)**	**0.092**	**0.015**	**0.670-0.830**	**This study**
**IK consortium**	**Starch (0.0)**	**0.022**	**0.004**	**0.210-0.275**	**This study**

*NA*: not available

1Values are estimated using the amounts of degraded RDX over a specific time period and the final OD (optical density) value of microbial growth and these values were obtained using a general calculation formula by assuming that all OD values were obtained at the same wavelength.

**Table 2:** Relative abundances of the major genus groups (%) that were detected in this study.

OTU ID	Accession number	Phylum/Class	Genera	Initial^1^	S0.0^2^	S2.5^3^	S5.0^4^
RCS1–RCS5	KR779830-KR779834	Firmicutes/ Bacilli	*Sporolactobacillus*	0.01	31.6	49.8	46.2
RCS6–RCS10	KR779835-KR779839	Firmicutes/ Clostridia	*Clostridium*	0.02	48.2	42.6	38.4
RCS11–RCS12	KR779840-KR779841	Firmicutes/ Bacilli	*Paenibacillus*	0.04	11.8	2.71	2.04
RCS13–RCS14	KR779842-KR779843	Proteobacteria/ Gamma	*Pseudomonas*	0.14	0.14	0.06	2.14
RCS15–RCS16	KR779844-KR779845	Firmicutes/ Negativicutes	*Sporotaelea*	0.00	0.01	0.01	1.13

^1^Initial: Soil initially used for the 1st enrichment

^2^S0.0: Microcosm with RDX in the absence of starch

^3^S2.5: Microcosm with RDX in the presence of 2.5 g/l starch

^4^S5.0: Microcosm with RDX in the presence of 5.0 g/l starch
