# Supplementary material for: A Sporolactobacillus-, Clostridium-, and Paenibacillus- Dominant Microbial Consortium Improved Anaerobic RDX Detoxification by Starch Addition
Source: J Microbiol Biotechnol. 2020 Mar 9;30(6):839–47. doi: 10.4014/jmb.1910.10034 (PMC9728379; doi:10.4014/jmb.1910.10034)
Supplement: Supplementary file 1 [file JMB-30-6-839-supple.pdf]

J. Microbiol. Biotechnol.
https://doi.org/10.4014/jmb.1910.10034

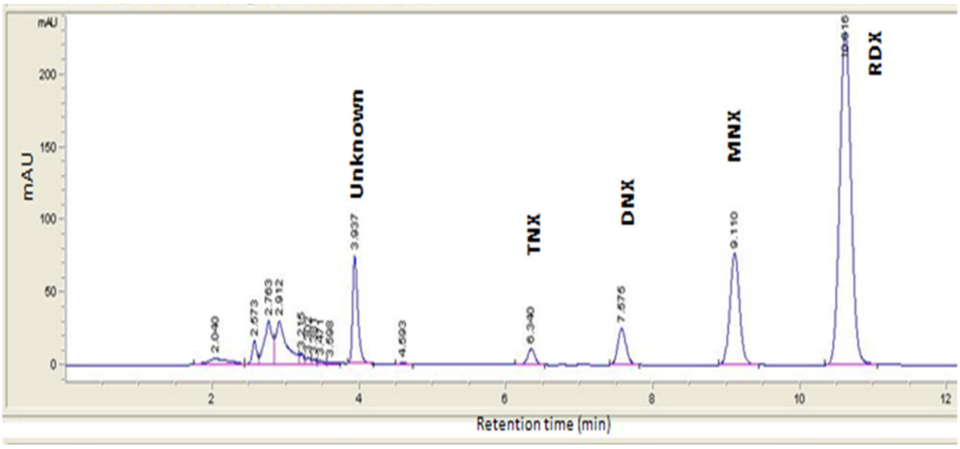

Fig. S1. HPLC chromatograms showing the formation of MNX, DNX, TNX, and an unknown metabolite during the microbial degradation of RDX after 5 days incubation.

Table S1

Scheme of microcosm experiments.

Experiment	Variants	Initial RDX conc. (mM)	Starch conc. (g/L)
1st enrichment	No RDX	0.000	2.5
	S0.0	0.052	0.0
	S1.0	0.056	1.0
	S2.5	0.055	2.5
	S5.0	0.055	5.0
	S7.5	0.058	7.5
	Autoclaved	0.056	0.0
	S2.5-autoclaved	0.059	2.5
	No microbes	0.052	0.0
2nd enrichment	S0.0	0.068	0.0
	S2.5	0.068	2.5
	S5.0	0.068	5.0
	Autoclaved	0.068	0.0
3rd enrichment	S0.0	0.089	0.0
	S2.5	0.091	2.5
	S5.0	0.089	5.0
	Autoclaved	0.093	0.0
